# Occurrence, Removal, and Mass Balance of Polycyclic Aromatic Hydrocarbons and Their Derivatives in Wastewater Treatment Plants in Northeast China

**DOI:** 10.3390/toxics9040076

**Published:** 2021-04-02

**Authors:** Rashid Mohammed, Zi-Feng Zhang, Chao Jiang, Ying-Hua Hu, Li-Yan Liu, Wan-Li Ma, Wei-Wei Song, Anatoly Nikolaev, Roland Kallenborn, Yi-Fan Li

**Affiliations:** 1 International Joint Research Center for Persistent Toxic Substances (IJRC-PTS), State Key Laboratory of Urban Water Resource and Environment, Harbin Institute of Technology (HIT), Harbin 150090, China; m13045102069@163.com (R.M.); liuliyan@hit.edu.cn (L.-Y.L.); mawanli@hit.edu.cn (W.-L.M.); songweiwei@hit.edu.cn (W.-W.S.); roland.kallenborn@nmbu.no (R.K.); 2 International Joint Research Center for Arctic Environment and Ecosystem (IJRC-AEE), Polar Academy, School of Environment, Harbin Institute of Technology (HIT), Harbin 150090, China; 3 Heilongjiang Provincial Key Laboratory of Polar Environment and Ecosystem (HPKL-PEE), Harbin Institute of Technology (HIT), Harbin 150090, China; 4 Heilongjiang Institute of Labor Hygiene and Occupational Diseases, Harbin 150028, China; sey53969319@126.com (C.J.); slinea88@163.com (Y.-H.H.); 5 Institute of Natural Sciences, North-Eastern Federal University, 677000 Yakutsk, Russia; an.nikolaev@s-vfu.ru; 6 Faculty of Chemistry, Biotechnology & Food Sciences (KBM), Norwegian University of Life Sciences (NMBU), 1432 Ås, Norway; 7 IJRC-PTS-NA, Toronto, ON M2N 6X9, Canada

**Keywords:** PAHs, wastewater treatment plant, model prediction, ecological risk assessment

## Abstract

Polycyclic aromatic hydrocarbons (PAHs), 33 methylated PAHs (Me-PAHs), and 14 nitrated PAHs (NPAHs) were measured in wastewater treatment plants (WWTPs) to study the removal efficiency of these compounds through the WWTPs, as well as their source appointment and potential risk in the effluent. The concentrations of ∑PAHs, ∑Me-PAHs, and ∑NPAHs were 2.01–8.91, 23.0–102, and 6.21–171 µg/L in the influent, and 0.17–1.37, 0.06–0.41 and 0.01–2.41 µg/L in the effluent, respectively. Simple Treat 4.0 and meta-regression methods were applied to calculate the removal efficiencies (REs) for the 63 PAHs and their derivatives in 10 WWTPs and the results were compared with the monitoring data. Overall, the ranges of REs were 55.3–95.4% predicated by the Simple Treat and 47.5–97.7% by the meta-regression. The results by diagnostic ratios and principal component analysis PCA showed that “mixed source” biomass, coal composition, and petroleum could be recognized to either petrogenic or pyrogenic sources. The risk assessment of the effluent was also evaluated, indicating that seven carcinogenic PAHs, Benzo[a]pyrene, Dibenz[a,h]anthracene, and Benzo(a)anthracene were major contributors to the toxics equivalency concentrations (TEQs) in the effluent of WWTPs, to which attention should be paid.

## 1. Introduction 

Polycyclic aromatic hydrocarbons (PAHs) are a type of contaminant with teratogenicity and carcinogenicity, which are discovered in various places, such as wastewater treatment [1]. Sixteen PAHs have been enlisted for priority pollutants by the United States Environmental Protection Agency (U.S. EPA) and seven carcinogenic polycyclic aromatic hydrocarbons were selected as possible human carcinogens [2]. Recently, some typical alternative aromatic hydrocarbons, particularly methylated polycyclic aromatic hydrocarbons (Me-PAHs) and nitrated polycyclic aromatic hydrocarbons (NPAHs), have been of great interest due to their higher toxicity than polycyclic aromatic hydrocarbons [3]. NPAHs have a higher molecular weight (MW), octanol-air partition coefficients (K_OA_), less water solubility (S), vapor pressure (V_P_), sorption partition (K_OC_), an octanol-water partition coefficient (K_ow_), than their related PAHs [4]. NPAHs and Me-PAHs may be released from similar sources of PAHs, for instance, incomplete combustion, vehicle engines, and spillage oil [5]. The derivatives can also be produced through the transformation of polycyclic aromatic hydrocarbons equivalent to different rings by chemical and biological processes [6]. NPAHs present in the aquatic environment could also be obtained from atmospheric deposition and urban drainage [7].

PAHs can enter wastewater treatment plants (WWTPs) after release from fuel spillage or atmospheric deposition to the system by domestic WWTPs, industrial discharge, or runoff through urban drainage [8]. Recent research reported that sewage water was the main origin of PAHs, and its derivatives in the rivers, receiving sewage water in Beijing [9]. The removal of polycyclic aromatic hydrocarbons within activated sludge can be related to three biotic or abiotic methods: (1) volatilization through abstraction governed by the physical characteristic of micro-pollutants by Henry’s law constant; (2) absorption into sludge as a result of hydrophobic reactions between pollutants and suspended solids that may lead to their removal through sludge waste; and (3) biotic transformation, including both complete degradation and transformation into byproducts, leading to the effective elimination of organic compounds [10]. Furthermore, the removal efficiency of WWTPs can face difficulties including their low aqueous solubility and bioavailability. PAHs are resistant to degradation and are difficult to be removed by conventional physicochemical techniques such as coagulation, flocculation, sedimentation, and filtration [11]. However, adsorption processes are efficient in removing persistent organic pollutants and, as a result to their efficiency, recyclability, and feasibility [12]. Therefore, adsorption techniques can be used to remove both hazardous and less soluble organic molecules, such as PAHs, from water [13].

The purposes of this research are: (1) to investigate the occurrence of PAHs, Me-PAHs, and NPAHs in the influents and effluents of the 10 WWTP treatment plants; (2) to find out the sources of PAHs, Me-PAHs, and NPAHs in WWTPs; and (3) to compute the impact of internal (chemical-related) and external (WWTP-related) factors on removal efficiencies (REs) of a group of compounds to obtain comprehensive and specific data on their removal in WWTPs. The results will provide basic information for the wastewater treatment plant to upgrade treatment processes and assess the water quality and risk assessment of the receiving river.

## 2. Materials and Methods

### 2.1. Samples Collection

**Figure 1:**
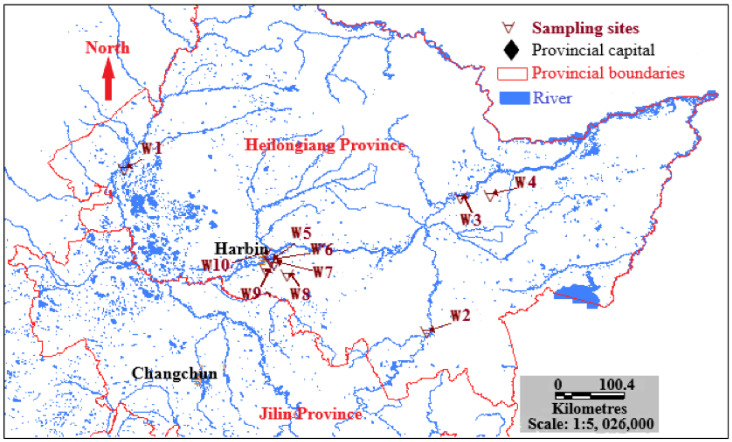
Sampling locations for 10 wastewater treatment plants in the Songhua River Basin, Northeast China.

Ten urban wastewater treatment plants (WWTP 1–10) were selected in the warm (June–July) and cold (October–November) seasons in 2017 along the Songhua River in Heilongjiang Province, China (Figure 1). The biological treatment effluent mainly flowed into the Songhua River. To examine the regular variation with an influent and effluent concentration of PAHs, Me-PAHs, and NPAHs, 24 h influent wastewater samples were collected from the 10 WWTPs. Raw influent was collected with the ISCO 3700 composite autosampler (Teledyne ISCO, Lincoln, NE, USA) for 24-h, compositing 24 subsamples of ~200 mL in each collection vessel to form a homogeneous sample. The effluent and sludge samples were collected using sampling tools. To reduce the uncertainty related to sampling, the samples were collected three times throughout a 24-h period, resulting in 3 samples being collected in one day. All the collected wastewater samples were kept in 1 L glass bottles. After collection, samples were sent to the laboratory at a low temperature of 4 °C within 24 h to avoid microbial degradation. Basic information for the ten WWTPs are presented in Table S1.

### 2.2. Pretreatment and Instrumental Analysis

Due to the high concentrations of the target compounds, the influent samples were processed by diluting 50 to 500 mL using purified water. The 500 mL effluent samples were processed without dilution, and all wastewater samples were unfiltered. The target compounds were extracted by solid-phase extraction (SPE) as described previously [14]. C18 tandem linked with HLB cartridges (500 mg 6 cc^−1^) (Waters–MILFORD–MA–USA) was noticed of DCM with 5 mL and 5 mL of MeOH, accompanied by ultrapure water (5 mL) at a rate of approximately 1 mL.min^−1^. After that, water samples (500 mL) were loaded at a rate of about 5 mL min^−1^. Then drying for 60 min with a gentle stream of N2, the SPE cartridges were fully clarified from the sorbent as follows: (i) into 15 mL tubes with 7 mL DCM and (ii) into 7 mL MeOH at a flow rate of 1 mL/min. A gentle stream of N2, around 1 mL, was used to extract the extracts, and the solvent was changed to 1 mL with toluene until being shifted to 1.5 mL.

The detection of PAHs was performed by use of Agilent 7890A-7000B gas chromatography-tandem triple-quadrupole mass spectrometry applied to an EI ion source (GC-EI-MS/MS). Agilent 19091J-433E (30 m × 250 µm × 0.25 µm) HP-5MS chromatographic column was employed in GC, and Multiple Reaction Monitoring (MRM) chromatogram. The parameters were as follows: (i) inlet temperature was 320 °C for PAHs, 280 °C for Me, N-PAHs; (ii) the injection pulse pressure was 40 psi until it reached 0.8 min; (iii) one microliter of the aliquot was injected in with pulsed splitless mode; (iv) then the flow rate was 1 mL/min; (v) while the gas saver was 20 mL min^−1^; after 3 min, (vi) afterward the septum purge flow 3 mL min^−1^; and finally (vii) the purge flow to split vent 50 mL min^−1^ at 1.2 min. All the transition collision energy and retention time parameters are listed in Table S3 and Figures S1–S3.

### 2.3. Quality Control/Quality Assurance (QC/QA)

Strict quality control (QC) procedures in this research were observed. Before being rinsed with distilled water, all glassware was thoroughly washed with soap and water, and after that, were heated in an oven at 120 °C for close to 6 h. Finally, they were rinse with hexane, as well as acetone, before use. To monitor method recovery and interferences, matrix-spiked samples and duplicates were analyzed, two levels of matrix-spiking (100 µL solution with 50 or 500 ng of native standards in acetone) were spiked into the influent and effluent samples for triplicates tests, waited for 30 min stabilization, and then followed the SPE procedures as above. The calibration curve of all the target analytes were prepared at concentrations ranging from 1 to 500 ng/mL (1, 5, 10, 20, 50, 100, 200, and 500 ng/mL) for native standards with 100 ng/mL of internal standards; as well as each sample type, and procedural blanks. The trace levels of the procedural blanks compared to actual samples can only be recognized at low-ring PAHs. Due to greater background in the procedural, blanks and a very high detecting rate (Nap) were excluded, and the reported concentrations in this research were blank-corrected. Analyzed recoveries angled from 70 to 125% in spiked matrix samples, and for duplicate samples, the coefficient of variation was below 20%. All of the surrogate average recoveries were between 66 and 105%, in all sample matrices, and for surrogate recovery, all analytical levels have been repaired.

The limits of detection (LODs) were calculated using a signal-to-noise (S/N) ratio of 3 for the standard solutions, while limits of quantification (LOQs) were determined by a S/N of 10. The data was kept, which were higher than the LOQs. The details are shown in Table S2, supplied in Supporting Information (SI).

### 2.4. Statistical Analyses

SPSS 20.0 statistical software packages were used to conduct statistical analyses. principal component analysis had been used to present relationships and trends within datasets. Moreover, the diagnostic ratio approach was utilized to discovered potential sources of PAHs and Me-PAHs in several functional areas, and the independent-sample *t*-test analysis was applied to check out the relationship between influent and effluent.

### 2.5. Modelling

Simple Treat 4.0 was used in this study to evaluate performance and removal efficiency in WWTP treatment plants. The physicochemical characteristics of the substances are displayed in Table S4. In Simple Treat, the classification of acid, neutral, and base depends on the ionized form of a chemical-based on pKa as well as pH., signifying the possibility of organic chemicals occurring in the ionized form [15,16]. Furthermore, only a classification regarding the Organization for Economic Co-Operation and Development (OECD) test outcomes were available, rates were appointed corresponding to the Simple Treat classification arrangement. The sludge loading rate was computed from the study’s specific sludge retention time (SRT) regarding the Struijs indications [17]. Additionally, the flow rate of 10 WWTPs was input immediately from our database. The efficiency of Simple Treat 4.0 was evaluated by the meta-regression model.

(1) $$V_{j}\frac{{\mathrm{dc}}_{j}}{\mathrm{dt}}=-K_{j}C_{j}V_{j}+\sum {\mathrm{ADV}}_{\mathrm{ij}}C_{i}+\sum {\mathrm{XCH}}_{\mathrm{ij}}C_{i}$$

(2) $$K_{j}=K_{\mathrm{biodeg}}1.072^{\left(T_{W}-288\right)}$$

where (C_i_) system flowing out via, water, air, or suspended solid as well as a concentration in the medium (i) when the chemical is transported from medium (i) to medium (j), (XCH_i__,j_) is the flow rate of media from a box (i) to box (j), reversible and diffusive; (C_j_) is the concentration inbox (j), (K_j_) is the first-order biodegradation rate, constant inbox (j); (ADV_i__,j_) is the flow rate of media from the box (i) to box (j). (T): time. (V_j_) is a volume of the box (j) [17].

### 2.6. Meta-Regression Model

Meta-regression model was estimated the average weighted effect size among all studies. This value had been transformed back into an overall (RE) removal efficiency of the chemicals present in our database systems. The (RE) mean weight was calculated once for the total number of effect sizes available, as follows:

(3) $$\mathrm{RR}=\ln\left(\frac{X_{\mathrm{ceff}}}{X_{\mathrm{cin}}}\right)$$

(4) $$\sigma^{2}\left(\mathrm{RR}\right)=\frac{{\left({\mathrm{SD}}_{\mathrm{ceff}}\right)}^{2}}{N_{\mathrm{ceff}}X_{{\mathrm{ceff}}^{2}}}+\frac{{\left({\mathrm{SD}}_{\mathrm{cin}}\right)}^{n}}{N_{\mathrm{cin}}X_{{\mathrm{cin}}^{2}}}$$

(5) $$\mathrm{RE}=1-e^{\left(\mathrm{RR}\right)}$$

where (SD_cin_) is the standard deviation of the influent concentration, (σ^2^) is sampling variance, (N_ceff_) is a number of samples for the effluent concentration, (X_cin_) is the mean of the influent concentration, (RR) is effect size response ratio, measured per wastewater treatment plant WWTP and compound, (SD_ceff_) is the standard deviation of the effluent concentration, (RE) is removal efficiency, (N_cin_) is the number of samples for the influent concentration and (X_ceff_) is mean of the effluent concentration [18].

### 2.7. Mass Loading

The mass loading (ML, g day^−1^) of the PAHs, Me-PAHs, and NPAHs in the influent sewage of the studied wastewater treatment plant and the discharge (g day^−1^) to the Songhua River during the sampling time was computed as follows [19]:

(6) $$\mathrm{ML}=C_{\mathrm{in}}\times \mathrm{FL}\times \mathrm{CF}$$

(7) $$\mathrm{Discharge}=C_{\mathrm{ef}}\times \mathrm{FL}\times \mathrm{CF}$$

where (C_in_) is the concentration of PAHs, Me-PAHs, and NPAHs detected for the influent, (FL) is the corresponding flow rate of the studied wastewater treatment plants (L day^−1^), (CF) is the conversion factor (10^−6^ mg ng^−1^), and (C_ef_) is the concentration of PAHs, Me-PAHs, and NPAHs detected for the final effluent.

### 2.8. Potential Cancers Risk Assessment

The potential cancer risk for (PAHs) was calculated as per Equations (8) and (9), by multiplying the concentration of each chemical compound by its corresponding (TEF) value [20]. ∑16 PAHs, total carcinogenic potency was calculated by summing the BaP- equivalent concentration of all compounds [21].

(8) $$\sum {\mathrm{BaP}}_{\mathrm{eq}}=\sum {\mathrm{PAHs}}_{i}\times {\mathrm{TEF}}_{i}$$

(9) $${\mathrm{TEQC}}^{\mathrm{CARC}}=\sum C_{i}\times {\mathrm{TEF}}_{i}$$

where (TEF) is the toxic equivalent factor provided, (Bap_eq_) is the carcinogenic potency of a congener evaluated dependent on (BaP_eq_) concentration, (C_i_) is the concentration (µg/L), (TEQ) represents toxic equivalence quotient [22,23].

## 3. Results

### 3.1. Occurrence and Profiles of PAHs, Me-PAHs, and NPAHs

#### 3.1.1. PAHs, Me-PAHs, and NPAHs in the Influent

The 16 PAHs, 33 Me-PAHs, and 14 NPAHs were detected in the influent and effluent of the 10 WWTPs. The total concentrations were from 2.01 to 8.91 µg/L, with a mean of 4.58 µg/L, for PAHs, from 23.0 to 102 µg/L, with a mean of 46.6 µg/L for Me-PAHs, and from 6.21 to 171 µg/L, with a mean of 47.3 µg/L, for NPAHs (Table 1). The total concentration of NPAHs was greater than PAHs and Me-PAHs, which was consistent with these observed in a preceding study [24].

Associated with the analyzed PAHs, Nap was the most abundant compound in influent samples, accounting for about 32% of the total PAHs concentration, followed by Phe, Acy, and Flu. The most abundance for Nap might be because Nap is the only one used for the production of both dyes and moth-killer products, which have been widely applied in the textile industry and people’s daily lives. Among investigated Me-PAHs, 2-MNAP, 1-MNAP, and 1,3-DMNAP were the major compounds in influent. The most abundant 2-MNAP displayed 14% of the total Me-PAHs concentration. The higher concentrations of 2-MNAP in the influent of the WWTPs have been reported [5,25]. For NPAHs, 2-N, 2-NAN, and 9,10-DNAN were the most abundant in influent, with 2-N representing 36% of the total concentration.

In general, compounds with low molecular weight, considered more toxic than those with high molecular weight, were prevailing in PAHs, Me-PAHs, and NPAHs in influent WWTPs. On average averagely, low molecular weight PAHs, Me-PAHs, and NPAHs concentrations were 3.13, 36.8, and 47.3 µg/L, respectively, and high molecular weight PAHs and Me-PAHs concentrations were 1.44 and 10.2 µg/L, respectively.

#### 3.1.2. PAHs, Me-PAHs, and NPAHs in the Effluent

Concentrations of the target compounds in the effluent of the ten WWTPs are presented in Table 1. Concentrations of the total chosen PAHs, Me-PAHs, and NPAHs detected through the sampling duration were in the variety of 0.17–1.37, 0.06–0.41, and 0.01–2.41 µg/L, with means of 0.54, 0.18, and 0.30 µg/L, respectively. Phe and NaP were the dominant compounds for PAHs, with median concentrations of 0.11 and 0.14 µg/L, respectively. Moreover, 2-MNAP and 1,6-DMNAP were the predominant compounds for Me-PAHs, with median concentrations of 0.03 and 0.01 µg/L. Among all NPAH compounds, 2-NAN, 4-NBP, and 9,10-DNAN were the prevalent ones, with median concentrations of 0.02 µg/L.

It was interesting to notice that PAHs became higher than Me-PAHs and NPAHs in the effluent, which probably resulted from their higher log K_OW_ and polarity values; the log K_OW_ of the investigated Me-PAHs and NPAHs were generally lower than those of PAHs. From one aspect, the compound was removed by adsorption, resulting in the lower removal efficiencies of the SPAHs and high removal of PAHs [24]. With a relative percentage of 25% for Phe, 18% for 2-MNAP, and 23% for 2-NAN. The strong volatility and biodegradability of Me-PAHs might also result in a low concentration. This change in relative contribution recommended that the removal efficiency of PAHs was much greater than Me-PAHs and NPAHs in the effluent of the WWTP. In effluent, the average concentrations were 0.39, 0.11, and 0.30 µg/L for LMW PAHs, Me-PAHs, and NPAHs, whereas 0.15 and 0.06 µg/L for HMW PAHs and Me-PAHs, respectively.

#### 3.1.3. Comparison of PAHs, Me-PAHs, and NPAHs in Sewage Worldwide

Comparisons of concentrations for PAHs, Me-PAHs, and NPAHs in influent and effluent of WWTPs worldwide are displayed in Table 2. The level of PAHs in this study was much lower than those in Maresme, Catalonia, Spain (14.29 µg/L) [26], Hefei City, situated beside Nanfei River, China (5.76 µg/L) [27], and Heraklion, Greece (11.07 µg/L) [28], but higher than the level in Hong Kong, China (0.30 µg/L) [29], Tai’an, China (1.16 µg/L) [30], Zhejiang, China (0.45 µg/L) [31], Guangzhou, China (0.93 µg/L) [8], Harbin, China (4.08 µg/L) [32], Jerez de la Frontera, Spain (1.92 µg/L) [33], Heraklion, Crete, south Greece (0.79 µg/L) [34], Higashi-Hiroshima City, Japan (0.219 µg/L)[35]. The degree of Me-PAHs in this research was greater than the Northwest of Beijing (0.22 µg/L) [36]. Daegu, Korea (1.35 µg/L) [37]. Furthermore, NPAHs in this study were also much greater than the southeast of Shandong China (1.19 µg/L) [38]. PAHs in the influents of the WWTPs were impacted by many factors, such as the proportion of industrial wastewater, local manufacturing, and rubber companies, machinery production [39]. Generally, the concentrations of all individual and total PAHs, Me-PAHs, and NPAHs decreased along with the treatment processes. The concentrations of total PAHs, Me-PAHs, and NPAHs in the effluent were with different researches as well. The degree of PAHs in this study were greater than those of Hong Kong, China (0.02 µg/L) [29], Hiroshima City (0.043 µg/L)[35] Tai’an City China (0.13 µg/L)[30], Zhejiang Province, China (0.01 µg/L) [31], Guangzhou, China (0.19 µg/L) [8], Nakdong river Korea (0.44 µg/L) [37], Jerez de la Frontera, Spain (0.50 µg/L) [33], however, lower than those of Hefei City, China (2.24 µg/L) [27], Harbin, Northeast China (0.86 µg/L) [32], Maresme, Catalonia, Spain (3.91 µg/L) [26], Thessaloniki, northern Greece (5.64 µg/L) [28]. The degree of Me-PAHs in the effluent in this research was greater than northwest of Beijing (0.06 µg/L) [36]. NPAHs in effluent in this study were also much higher than the southeast of Shandong China (0.24 µg/L) [38]. The stage of PAHs in effluent had a significant effect on microbial degradation, weather conditions [33]. There were strong correlations between the influent and effluent, PAHs (R^2^ = 0.83, *p* < 0.01), Me-PAHs (R^2^ = 0.51, *p* < 0.01), but the weak correlation for NPAHs (R^2^ = 0.13, *p* < 0.01), as shown in Figure S4, which, signified that higher concentrations in influents often led to (very) higher levels in effluents.

### 3.2. Removal Efficiencies of PAHs, Me-PAHs, and NPAHs by Model Prediction

**Figure 2:**
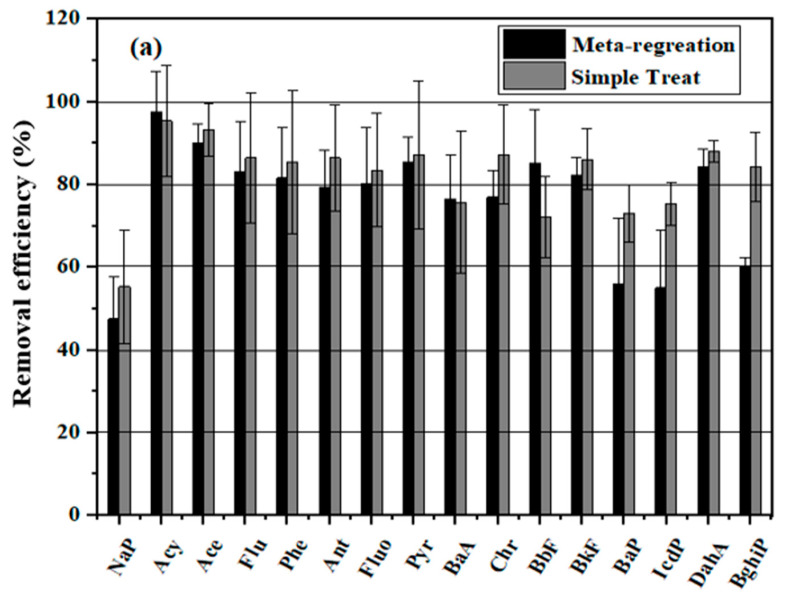

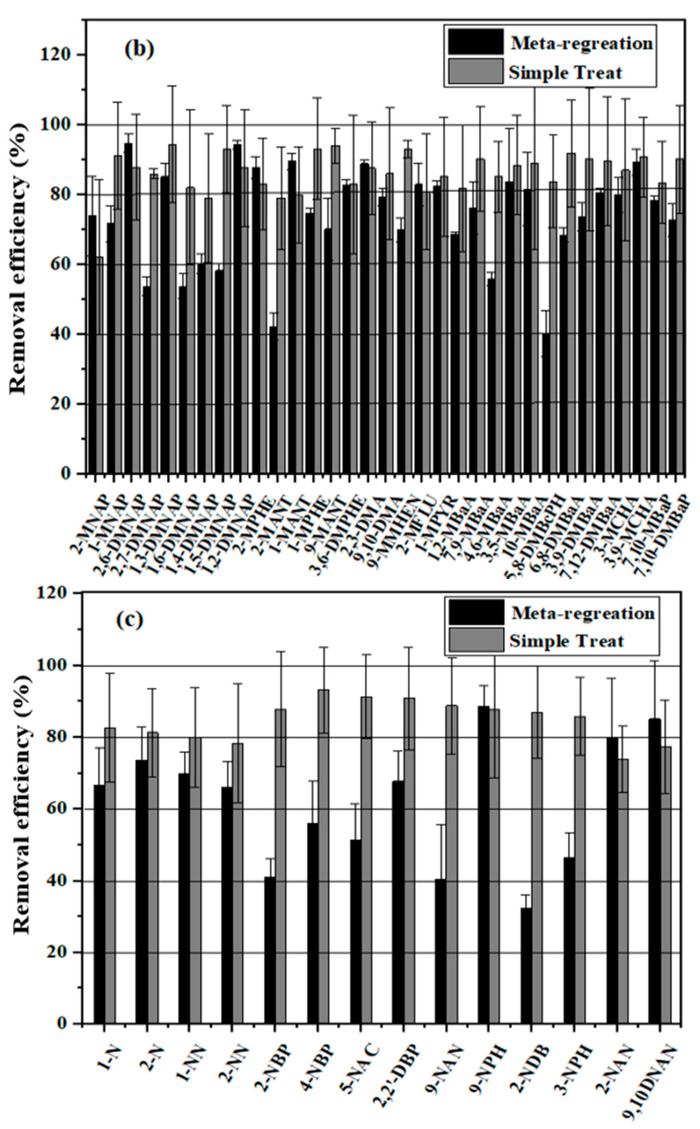
The removal efficiency of PAHs (**a**), Me-PAHs (**b**), and NPAHs (**c**) at 10 WWTP, prediction by Simple Treat 4.0 and meta-regression (mean ± SD).

**Figure 3:**
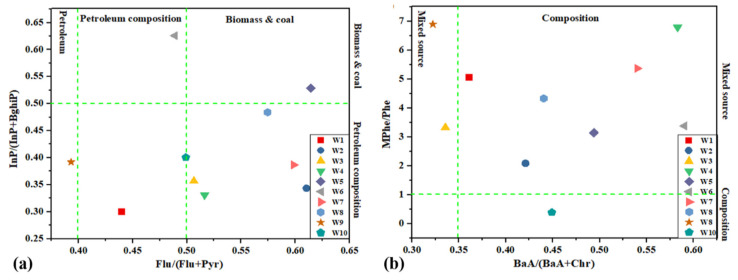
Plots showing PAHs and Me-PAHs isomeric ratios (**a**) Flu/(Flu+Pyr) vs. InP/(InP + BghiP) and (**b**) BaA/(BaA + Chr) vs. (MPhe/Phe).

In this research, both models, Simple Treat 4.0 and Meta-Regression, were applied to estimate removal efficiencies of PAHs, Me-PAHs, and NPAHs in the ten wastewater treatment plants, and the detected mean removal efficiencies, as displayed in Figure 2. Corresponding to Simple Treat 4.0, mean removal efficiencies of PAHs, Me-PAHs, and NPAHs ranged from 55.3% (NaP) to 95.4% (Acy) (Figure 2a), from 62.2% (2-MNAP) to 94% (9-MANT) (Figure 2b), and from 73% (2-NAN) to 91% (5-NAC) (Figure 2c), respectively. The mean removal efficiencies of PAHs, Me-PAHs, and NPAHs predicted by the meta-regression model ranged from 47.5% for NaP to 97.7% for Acy (Figure 3a), from 40.4% for 5,8-DMBcPH to 94.8% for 2,6-DMNAP, and from 32.2% for 2-NDB (Figure 3b) to 85.0% for 9,10-DNAN (Figure 2c). The model results recommended that the 2–3 ring PAHs (Acy, Ace) can be removed more effectively than NaP, Phe, and Ant. The volatility of NaP could be an attainable description for the excessive NAP concentration in this effluent [40]. The 4–6 ring (BbF, BaP, and IcdP) had been removed, much less contrasted to BaA, Chr, BkF, DahA, and BghiP. The less removal effectively for LMW PAHs, Me-PAHs, and NPAHs in contrast to HMW PAHs and Me-PAHs at WWTPs signifies the importance of biodegradation and evaporation of PAHs fraction. In contrast, high molecular weight PAHs are less soluble and volatile, so they are mainly associated with particles and less available for degradation in the water [41].

Compounds with log Kow > 5 were anticipated to be eliminated through sorption at competences between 60% and 65%, while for a compound with log Kow values between 3.6 and 5, lower removals are expecting 15–50% due to this mechanism [28]. Considering this mechanism, the Simple Treat and Meta-regression model were plotted against the compounds with log K_OW_ values, as explained in Figure S5, showing statistically significant correlations (R^2^ = 0.64, *p* < 0.01) by using Simple Treat and (R^2^ = 0.43, *p* < 0.01) by Meta-regression. This connection advocated that compounds with log K_OW_ > 5 had greater efficacious than the compound with log K_OW_ ˂ 5 in the elimination mechanism for PAHs. It can be concluded that estimations of two models had contrasted the removal effectivity of PAHs, Me-PAHs, and NPAHs; model predictions have the ability of standard process-based models to accurately predict the influence of definite parameters on removal efficiencies, which high and low removal efficiencies were reported, the physicochemical properties of the HMW PAHs, Me-PAHs, and NPAHs, their behavior during the treatment process appeared through removal efficiency. Furthermore, the removal efficiencies range from 55.3 to 95.4% detected by the Simple Treat and from 47.5 to 97.7% by meta-regression, in contrast, Simple Treat 4.0 was qualified to detect the removal competencies relatively (within a factor 4). In addition, Simple Treat displays actual WWTP conditions, with little physicochemical information was available and depends on the official wastewater operational.

### 3.3. Source Apportionment by Principal Component Analysis

The potential sources in this research were recognized by principal component analysis. Three factors had been extracted in WWTPs, with a total variance of 89.0%. The rotated component loadings are pointed out in Table S5. Factor PC1 principal component was consorting with 44.9% of the variance and mainly loading on BbF, BaA, BaP, BkF, and Chr. The BaA, Chr, and BaP are commonly used to provide facts about PAHs origin and sources in environmental samples, suggesting excessive loadings of a pyrolytic source [40]. PC2 revealed 30.6% of the variance and obtained excessive loading for IcdP, BghiP, and DahA, suggesting that the compounds have been released from gas and diesel vehicle emissions [42]. Eventually, PC3 (13.4% of the variance) was determent by NaP, Ace, Acy, and Flu, recommending that the prevalence of low molecular weight PAHs signifies the petrogenic source [22].

Corresponding to PCA analysis, there was a specific combination of PAHs as displayed in Table S5. Generally, there are several combinations of PAHs in wastewater (LMW 2,3 rings and HMW 4 to 6-rings); thus, we conditionally named PAHs sources in three factors as “mixed source”. Moreover, in every factor, the character of PAHs should be identified as both a pyrogenic or petrogenic source.

### 3.4. Source Apportionment by Diagnostic Ratios

The diagnostic ratios amongst various PAHs have been utilized extensively to distinguishing potential emission sources in the environment [37]. Consequently, characteristic ratios of Flu/Flu+Pyr, InP/InP+BghiP, and BaA/BaA+Chr, MPhe/Phe were computed to recognize PAHs and Me-PAHs in the present study (Figure 3a,b). The result confirmed that ratios of Flu/Flu+Pyr, InP/InP+BghiP were varied from 0.40 to 0.50 and 0.50 to 0.65, 0.25 to 0.50, and 0.50 to 0.65, recommend that mixture of biomass and coal composition and petroleum composition was a potential major source in the wastewater treatment plant (Figure 3a), and ratios of BaA/BaA+Chr, MPhe/Phe was ranged from 0.30 to 0.35 and 0.35 to 0.36, 0.30 to 1, and 1 to 7, respectively, point out that mixture of combined source and biomass and coal composition was a possible major source in the WWTP (Figure 3b). The outcomes from diagnostic ratios in his study confirmed that “mixed source” biomass and coal composition and petroleum have been the essential sources in the ten wastewater treatment plants, Heilongjiang province, North China.

### 3.5. Ecological Risk Assessment

The PAHs, Me-PAHs, and NPAHs were released from the WWTPs to the Songhua River, which was closely connected with the lives of the urban resident. The Toxic, carcinogenic equivalents (TEQs) value of the 10 WWTP effluent target pollutants was calculated. The TEQs, ∑PAHscarc, and ∑PAHs in different wastewater were pointed out in Table 3. As proven in the result, the TEQ concentrations of BaP, DahA, and BaA in all the 10 WWTPs had been much greater than other compounds. Accordingly, the pollution degree of HMW compounds needs to be given interest and considering greater exposure in this study. Moreover, as indicated from the results, the ∑PAHs_carc_ had been the principal contributor to TEQ. The TEQ in different 10 WWTPs were dissimilar: the higher TEQ as follows WWTP 4 (32.1 ng/L), WWTP 5 (29.2 ng/L), WWTP 6 (25.2 ng/L), and WWTP 3 (24.8 ng/L), respectively Table 3. Contrasted with the other researches, the effluent TEQBaP in this study was greater than the EU annual average environmental quality standard (AA-EQS = 0.17 ng/L) [32] and higher than that in effluent wastewater from Beijing (31.7 ng/L) [24], effluent wastewater concentrations from the city of Prato (8.3 ng/L) [25], effluent from two Italian Municipal WWTPs (7.8 ng/L) [43]. Furthermore, the potential carcinogenicity of PAHs in the WWTP effluent was very high compared to other researches, and it needs to be stressed.

## 4. Conclusions

In this research, the exposure degree of PAHs, Me-PAHs, and NPAHs in wastewater from Heilongjiang Province, Northeast China, had been explored. In the influent, the total average concentrations of ∑NPAHs (47.3 µg/L) had been higher than these of ∑Me-PAHs (46.6 µg/L) and ∑PAHs (4.58 µg/L) in contrast in effluent ∑PAHs (0.54 µg/L) had been greater than those of ∑Me-PAHs (0.18 µg/L), and ∑NPAHs (0.30 µg/L). The concentrations of Nap, 2-MNAP, and 2-N had been the highest among PAHs, Me-PAHs, and NPAHs, and concentrations for LMW had been greater than these for HMW. The Simple Treat model executed higher than the meta-regression model in predicting elimination efficiency. Regarding the Simple Treat model, Res, SRT, and biodegradability can remove the chemical compounds properly. Furthermore, the essential sources were mixed petrogenic and pyrolytic sources related to diagnostic ratios and principal component analysis. It was once observed that the TEQ concentrations of 7 carcinogenic PAHs in the effluent of wastewater had been greater than the EU (AA-EQS), which needs to be seriously taken into attention.

## Figures and Tables

**Table 1:** Concentrations (µg/L) of ∑16 PAHs, ∑33 Me-PAHs, and ∑14 NPAHs from 10 wastewater treatment plants (WWTPs).

Compound	Influent (µg/L)			Effluent (µg/L)		
	Range	Mean	Median	Range	Mean	Median
∑PAHs	2.01–8.91	4.58	3.97	0.17–1.37	0.54	0.48
LMW PAHs	1.24–6.05	3.13	2.63	0.13–1.01	0.39	0.34
HMW PAHs	0.75–2.85	1.44	1.33	0.04–0.35	0.15	0.13
LMW/HMW	1.65–2.12	2.17	1.97	3.25–2.90	2.6	2.61
∑Me-PAHs	23.0–102	46.6	38.5	0.06–0.41	0.18	0.16
LMW Me-PAHs	19.3–67.9	36.8	31.5	0.06–0.26	0.11	0.09
HMW Me-PAHs	3.76–34.6	10.22	7.16	BDL-0.15	0.06	0.06
LMW/HMW	1.96–5.13	3.60	4.37	118–1.74	1.82	1.54
∑NPAHs	6.21–171	47.3	31.0	0.01–2.41	0.30	0.76
LMW NPAHs	6.21–171	47.3	31.0	0.01–0.37	0.30	0.76

BDL, below the detection limit. PAHs, polycyclic aromatic hydrocarbons. Me-PAHs, Methylated polycyclic aromatic hydrocarbons. NPAHs, Nitrated polycyclic aromatic hydrocarbons. LMWPAHs/HMW PAHs = ∑2 to 3 rings/∑4 to 6 rings.

**Table 2:** The concentrations (µg/L) of the target compounds of different places worldwide.

Country	Sampling Sites	Concentrations of PAHs		N of PAHs	Reference
		Influent	Effluent		
China	Heilongjiang, Province	4.58	0.55	16 PAHs	This study
China	Hong Kong	0.30	0.02	16 PAHs	[29]
China	Tai’an City	1.16	0.13	16 PAHs	[30]
China	Zhejiang Province	0.45	0.01	16 PAHs	[31]
China	Hefei City	5.76	2.24	16 PAHs	[27]
China	Guangzhou, China	0.93	0.19	16 PAHs	[8]
China	Harbin, Northeast	4.08	0.86	16 PAHs	[32]
Spain	Maresme, Catalonia	14.29	3.91	16 PAHs	[26]
Korea	Daegu, Korea	1.35	0.44	16 PAHs	[37]
Spain	Jerez de la Frontera	1.92	0.50	10 PAHs	[33]
Greece	Heraklion, Crete, South	0.79	-	16 PAHs	[34]
Greece	Thessaloniki, northern	11.07	5.64	16 PAHs	[28]
China	Southeast of Shandong	1.19	0.24	5 NPAHs	[38]
Japan	Higashi-Hiroshima City	0.219	0.043	16 PAHs	[35]
China	Heilongjiang, Province	46.60	0.18	33 Me-PAHs	This study
China	Heilongjiang, Province	47.70	0.11	14 NPAHs	This study

**Table 3:** PAHs TEQ concentrations in different WWTPs µg/L.

PAHs	TEF	WWTP 1	WWTP 2	WWTP 3	WWTP 4	WWTP 5	WWTP 6	WWTP 7	WWTP 8	WWTP 9	WWTP 10
NaP	0.001	0.10	0.04	0.14	0.13	0.15	0.09	0.27	0.17	0.13	0.06
Acy	0.001	0.02	0.01	0.01	0.03	0.05	0.02	0.03	0.02	0.01	0.02
Ace	0.001	0.01	0.00	0.01	0.01	0.01	0.01	0.01	0.02	0.01	0.00
Flu	0.001	0.04	0.05	0.06	0.05	0.01	0.04	0.07	0.14	0.04	0.02
Phe	0.001	0.09	0.14	0.12	0.14	0.04	0.09	0.11	0.45	0.08	0.05
Ant	0.01	0.11	0.11	0.08	0.20	0.23	0.12	0.28	0.49	0.13	0.07
Fluo	0.001	0.01	0.01	0.02	0.04	0.02	0.02	0.05	0.09	0.01	0.02
Pyr	0.001	0.01	0.01	0.03	0.03	0.03	0.03	0.06	0.05	0.01	0.01
BaA	0.1	0.27	0.43	4.90	1.93	3.50	4.07	2.44	0.76	0.44	0.67
Chr	0.01	0.02	0.02	0.33	0.25	0.22	0.27	0.17	0.07	0.02	0.04
BbF	0.1	0.42	0.73	2.57	2.61	3.07	1.87	1.97	0.66	0.55	1.05
BkF	0.1	0.96	0.53	0.89	1.99	2.38	1.13	2.46	2.36	0.86	0.93
BaP	1	BDL	BDL	9.69	17.7	14.2	11.3	14.2	4.62	3.40	17.6
IcdP	0.1	BDL	BDL	0.86	1.39	0.86	0.73	0.56	BDL	BDL	BDL
DahA	1	BDL	BDL	4.97	5.37	4.12	5.18	BDL	BDL	BDL	BDL
BghiP	0.01	0.01	0.02	0.15	0.14	0.21	0.21	0.14	0.03	BDL	0.04
∑7 PAHs_carc_	2.41	1.68	1.72	24.2	31.2	28.4	24.6	21.8	8.49	5.28	20.3
∑ 16 PAHs	2.43	2.12	2.16	24.8	32.12	29.2	25.2	22.9	10.0	5.75	20.6

BDL: Below detection limit. ∑PAH_Scarc_: total polycyclic aromatic hydrocarbons carcinogenic including (BaA, Chr, BbF, BkF, BaP, InP and DahA).

## Data Availability

The data collected are property of our research center but will be made available by the corresponding author when requested.

## Supplementary Materials

The following are available online at https://www.mdpi.com/article/10.3390/toxics9040076/s1, Figure S1: MRM chromatogram of target PAHs, Figure S2: MRM chromatogram of target Me-PAHs, Figure S3: MRM chromatogram of target NPAHs, Figure S4: Scatterplot influent vs effluent of ∑PAHs, ∑Me-PAHs and ∑NPAH, Figure S5: Percent removal efficiency of PAHs, Model Simple Treat (a) Model Meata-regression (b) versus Log Know, Table S1: Basic information for the ten WWTPs, Table S2: Determined of LOD ng/mL and LOQ ng/Ml, Table S3: GC-MS/MS detection parameters of target PAHs, including the optimized retention time, transitions and collision energy (CE), Table S4: PAHs, Me-PAHs and NPAHs physiochemical properties. Re [4,44], Table S5: Factor pattern of PCA for PAHs in 10 WWTPs, China.
