# Supplementary material for: Occurrence, Removal, and Mass Balance of Polycyclic Aromatic Hydrocarbons and Their Derivatives in Wastewater Treatment Plants in Northeast China
Source: Toxics. 2021 Apr 2;9(4):76. doi: 10.3390/toxics9040076 (PMC8066243; doi:10.3390/toxics9040076)
Supplement: Supplementary file 1 [file toxics-09-00076-s001.pdf]

toxics

MDPI

Article

Supplementary Material: Occurrence, Removal, and Mass Balance of Polycyclic Aromatic Hydrocarbons and Their Derivatives in Wastewater Treatment Plants in Northeast China

Rashid Mohammed, Zi-Feng Zhang, Chao Jiang, Ying-Hua Hu, Li-Yan Liu, Wan-Li Ma, Wei-Wei Song, Anatoly Nikolaev, Roland Kallenborn and Yi-Fan Li

Chemicals and Reagents

All the solvents used in the experiments were high-performance liquid chromatography (HPLC)-grade quality. Dichloromethane (DCM), methanol (MeOH), and toluene were purchased from Fisher Scientific (Fair Lawn, New Jersey, USA). Pure (> 18 MΩ-cm R) reagent water was prepared by a Milli-Q system (Millipore, Billerica, MA, USA).

All the PAHs mixture standards were purchased from AccuStandard (New Haven, CT, USA). The 98% pure deuterium-labeled 16 PAHs mixture standards were purchased from AccuStandard. 2-Methylnaphthalene-d_{10}, 1-Methylnaphthalene-d_{10}, 9-Methylanthracene-d_{12}, 1-Nitronaphthalene-d_7 and 9-Nitroanthracene-d_9 were purchased from Chiron (Akershus, Norway).

Table S1. Basic information for the ten WWTPs.

Site	Longitude	Latitude	Start Time	Type of Treatment	Design Capacity (m^3/d)	Source
W1	123	47.2	2003	Activated Sludge Process	61,600	Domestic and industrial
W2	129	44.6	2003	Anoxic/Oxic Process	100,000	Domestic and industrial
W3	130	46.8	2006	Sequencing Batch Reactors Process	60,000	Domestic and industrial
W4	130	46.8	2010	Sequencing Batch Reactors Process	44,000	Domestic
W5	126	45.7	2011	Anoxic/Oxic Process	38,000	Domestic
W6	126	45.5	2009	Anoxic/Oxic Process	64,000	Domestic and industrial
W7	126	45.6	2013	Cyclic Activated Sludge System	46,000	Domestic
W8	126	45.8	2005	Anoxic/Oxic Process	286,400	Domestic and industrial
W9	126	45.8	2003	Anoxic/Oxic Process	143,700	Domestic and industrial
W10	126	45.9	2011	Cyclic Activated Sludge System	100,100	Domestic and industrial

W1 to W10, represents wastewater treatment plants sites.

Table S2. Determined of LOD ng/mL and LOQ ng/mL.

Analytes	Abbreviation	LOD ng/mL	LOQ ng/mL
R-PAHs			
Naphthalene	NaP	0.0052	0.018
Acenaphthylene	Acy	0.0524	0.1752
Acenaphthene	Ace	0.2224	0.7408
Fluorene	Flu	0.048	0.16
Phenanthrene	Phe	0.0144	0.0476
Anthracene	Ant	0.0148	0.0496

Fluoranthene	Fluo	0.0052	0.0172
Pyrene	Pyr	0.0044	0.0144
Benz(a)anthracene	BaA	0.0248	0.0828
Chrysene	Chr	0.024	0.08
Benzo(b)luoranthene	BbF	0.028	0.0928
Benzo(k)fluoranthene	BkF	0.0268	0.0888
Benzo(a)pyrene	BaP	0.4688	1.5624
Indeno(1,2,3-cd)pyrene	IcdP	0.0716	0.2388
Dibenz(a,h)anthracene	DahA	0.0152	0.0512
Benzo(g,h,i)perylene	BghiP	0.0148	0.0488
Me-PAHs			
2-Methylnaphthalene	2-MNAP	0.0052	0.0172
1-Methylnaphthalene	1-MNAP	0.0204	0.0676
2,6-Dimethylnaphthalene	2,6-DMNAP	0.004	0.0132
2,7-Dimethylnaphthalene	2,7-DMNAP	0.0152	0.0508
1,3-Dimethylnaphthalene	1,3-DMNAP	0.0124	0.042
1,6-Dimethylnaphthalene	1,6-DMNAP	0.0168	0.0564
1,4-Dimethylnaphthalene	1,4-DMNAP	0.0136	0.0456
1,5-Dimethylnaphthalene	1,5-DMNAP	0.0148	0.0496
1,2-Dimethylnaphthalene	1,2-DMNAP	0.0092	0.0304
2-Methylphenanthrene	2-MPHE	0.0224	0.0752
2-Methylanthracene	2-MANT	0.048	0.1604
1-Methylanthracene	1-MANT	0.0192	0.0644
1-Methylphenanthrene	1-MPHE	0.018	0.0604
9-Methylanthracene	9-MANT	0.0392	0.13
3,6-Dimethylphenanthrene	3,6-DMPHE	0.0784	0.2616
2,3-Dimethylanthracene	2,3-DMA	0.0864	0.288
9,10-Dimethylanthracene	9,10-DMA	0.5084	1.6948
9-Methyl-9-phenylfluorene	9-MMHEN	0.0036	0.012
2-Methylfluoranthene	2-MFLU	0.0068	0.022
1-Methylpyrene	1-MPYR	0.0076	0.0248
1-Methylbenz(a)anthracene	1-MBaA	0.0044	0.0144
7-Methylbenz(a)anthracene	7-MBaA	0.0036	0.0112
3-Methylbenz(a)anthracene	3-MBaA	0.0024	0.0092
10-Methylbenz(a)anthracene	10-MBaA	0.1256	0.4184
5,8-Dimethylbenzo(c)phenanthrene	5,8-DMBcPH	0.026	0.0868
6,8-Dimethylbenz(a)anthracene	6,8-DMBaA	0.0448	0.1488
3,9-Dimethylbenz(a)anthracene	3,9-DMBaA	0.0396	0.1324
7,12-Dimethylbenz(a)anthracene	7,12-DMBaA	0.2832	0.9432
3-Methylcholanthrene	3-MCHA	0.4412	1.4704
9-Methylbenzo(a)pyrene	9-MBaP	0.0724	0.2416
7-Methylbenzo(a)pyrene	7-MBaP	0.2144	0.7144
7,10-Dimethylbenzo(a)pyrene	7,10-DMBaP	0.4168	1.3888
N-OH-PAHs			
1-Naphthol	1-N	0.0608	0.2024
2-Naphthol	2-N	0.078	0.2604
1-Nitronaphthalene	1-NN	0.1096	0.3648
2-Nitronaphthalene	2-NN	0.0436	0.1456
2-Nitrobiphenyl	2-NBP	0.0256	0.0856
4-Nitrobiphenyl	4-NBP	0.0172	0.058
5-Nitroacenaphthene	5-NAC	0.1864	0.6212

2,2′-Dinitrobiphenyl	2,2′-DBP	0.0328	0.1096
9-Nitroanthracene	9-NAN	0.268	0.8928
9-Nitrophenanthrene	9-NPH	0.5	1.6668
2-Nitrodibenzothiophene	2-NDB	0.0608	0.202
3-Nitrophenanthrene	3-NPH	0.142	0.474
2-Nitroanthracene	2-NAN	0.7896	2.6316
9,10-Dinitroanthracene	9,10-DNAN	0.4616	1.5384

LOD, limits of detection. LOQ, limits of quantification. PAHs, polycyclic aromatic hydrocarbons.

Table S3. GC-MS/MS detection parameters of target PAHs, including the optimized retention time, transitions and collision energy (CE).

Analytes	Retention Time (min)	Transition 1 (m/z)	CE 1 (eV)	Transition 2 (m/z)	CE 2 (eV)
Naphthalene	5.077	128→102	20	128→127	20
Naphthalene-D8	5.020	136→108	10	136→84	15
Acenaphthylene	7.142	152→150	25	152→151	25
Acenaphthylene-D8	7.098	160→158	25	160→132	30
Acenaphthene	7.399	153→152	25	153→151	25
Acenaphthene-D10	7.328	162→160	30	162→158	30
Fluorene	8.213	166→165	25	165→163	30
Fluorene-D10	8.134	176→174	20	176→172	40
Phenanthrene	10.630	178→176	25	178→152	25
Phenanthrene-D10	10.508	188→160	30	188→184	40
Anthracene	10.793	178→176	25	178→152	25
Fluoranthene	15.356	202→200	35	202→201	25
Fluoranthene-D10	15.261	212→208	40	212→210	30
Pyrene	16.053	202→200	35	202→201	25
Pyrene-D10	15.965	212→208	40	212→210	40
Benz(a)anthracene	19.670	228→226	30	228→202	30
Benz(a)anthracene-D12	19.589	240→236	40	240→212	40
Chrysene	19.779	228→226	30	228→202	30
Chrysene-D12	19.681	240→236	40	240→212	30
Benzo[b]fluoranthene	22.325	252→250	30	252→226	25
Benzo[b]fluoranthene-D12	22.249	264→260	40	264→236	40
Benzo[k]fluoranthene	22.389	252→250	30	252→226	25
Benzo[k]Fluoranthene-D12	22.312	264→260	40	264→236	40
Benzo[a]pyrene	22.996	252→250	30	252→226	25
Benzop[a]pyrene-D12	22.921	264→260	40	264→236	40
Indeno[1,2,3-cd]pyrene	25.331	276→274	45	276→272	50
Indeno[1,2,3-cd]pyrene-D12	25.253	288→284	40	288→286	40
Dibenz(a,h)anthracene	25.410	278→274	55	278→276	50
Dibenzo[a,h]anthracene-D14	25.309	292→288	40	292→290	30
Benzo[g,h,i]perylene	25.914	276→274	45	276→272	50
Benzo[g,h,i]perylene-D12	25.820	288→284	40	288→286	30
Me-PAHs					
2-Methylnaphthalene	9.245	141→115	20	142→141	20
2-Methylnaphthalene-d10	9.050	150→122	30	152→122	35
1-Methylnaphthalene	9.548	141→115	20	142→141	20
1-Methylnaphthalene-d10	9.354	152→150	20	150→122	20

2,6-Dimethylnaphthalene	11.156	156→141	20	156→115	40
2,7-Dimethylnaphthalene	11.165	156→141	15	141→115	20
1,3-Dimethylnaphthalene	11.410	141→115	20	156→141	20
1,6-Dimethylnaphthalene	11.459	156→141	20	141→115	20
1,4-Dimethylnaphthalene	11.732	141→115	20	156→141	20
1,5-Dimethylnaphthalene	11.783	141→115	20	156→141	20
1,2-Dimethylnaphthalene	11.982	141→115	25	156→141	15
2-Methylphenanthrene	19.414	192→191	20	191→189	30
2-Methylanthracene	19.680	192→191	20	192→189	40
1-Methylanthracene	19.964	192→191	20	192→189	40
1-Methylphenanthrene	20.029	192→191	20	191→189	30
9-Methylanthracene	21.015	192→191	20	191→189	30
9-Methylanthracene-d12	20.753	204→202	25	202→198	30
3,6-Dimethylphenanthrene	22.588	206→191	20	206→189	45
2,3-Dimethylanthracene	25.113	206→191	20	206→189	45
9,10-Dimethylanthracene	27.022	206→191	20	191→189	30
9-Methyl-9-phenylfluorene	27.197	241→239	35	256→241	15
2-Methylfluoranthene	28.001	216→215	25	215→213	40
1-Methylpyrene	29.192	216→215	25	215→189	30
1,2-Methylbenz[a]anthracene	31.975	242→241	20	242→239	50
7,9-Methylbenz[a]anthracene	32.038	242→241	20	242→239	45
4,6-Methylbenz[a]anthracene	32.100	242→241	20	242→239	45
3,5-Methylbenz[a]anthracene	32.218	242→241	20	242→239	50
10-Methylbenz[a]anthracene	32.453	242→241	20	242→239	45
5,8-Dimethylbenzo[c]phenanthrene	32.751	256→241	20	256→239	50
6,8-Dimethylbenz[a]anthracene	32.848	256→239	45	256→241	20
3,9-Dimethylbenz[a]anthracene	32.891	256→239	50	256→255	25
7,12-Dimethylbenz(a)anthracene	33.305	256→241	20	256→239	50
3-Methylcholanthrene	34.467	268→252	40	268→253	20
8,9-Methylbenzo[a]pyrene	34.512	266→265	20	266→263	50
7,10-Methylbenzo[a]pyrene	34.693	266→265	20	266→263	50
7,10-Dimethylbenzo[a]pyrene	35.722	280→265	20	280→264	35
NPAHs					
1-Naphthol	12.853	144→115	45	144→116	15
2-Naphthol	13.009	144→115	20	144→116	10
1-Nitronaphthalene	14.293	127→77	15	173→115	20
1-Nitronaphthalene-D7	14.254	180→122	35	134→82	20
2-Nitronaphthalene	14.881	173→115	10	127→77	20
2-Nitrobiphenyl	15.581	152→151	20	182→154	15
4-Nitrobiphenyl	18.356	199→169	10	199→141	20
5-Nitroacenaphthene	22.659	199→169	10	152→151	25
2,2′-Dinitrobiphenyl	24.842	198→115	20	168→139	25
9-Nitroanthracene	27.170	223→193	15	223→165	35
9-Nitroanthracene-D9	27.121	232→174	35	232→202	15
9-Nitrophenanthrene	28.492	177→176	15	223→165	25
2-Nitrodibenzothiophene	28.882	183→139	20	229→183	20
3-Nitrophenanthrene	29.133	223→165	15	223→177	20
2-Nitroanthracene	29.699	223→177	20	177→176	15

9,10-Dinitroanthracene	30.138	268→55	10	176→150	20

GC-MS/MS, Gas chromatography mass spectrometry. PAHs, polycyclic aromatic hydrocarbons. CE 1 and CE 2, collision energy.

Table S4. PAHs, Me-PAHs and NPAHs physiochemical properties. Re [1,2].

PAHs	RN	Formula	MW	Solubility mg/L	Log Kow	Log KoA	Log Koc
NaP	2	$C_{12}H_{12}$	128	320	3.29	6.23	2.97
Acy	3	$C_{12}H_{8}$	152	5.30	4.07	6.28	3.40
Ace	3	$C_{13}H_{10}$	154	3.93	3.92	6.58	3.66
Flu	3	$C_{10}H_{12}$	166	1.85	4.90	7.33	3.86
Phe	3	$C_{14}H_{10}$	178	1.24	4.15	7.09	4.15
Ant	3	$C_{14}H_{10}$	178	0.64	4.54	8.32	4.15
Fluo	4	$C_{16}H_{10}$	202	0.25	5.22	9.10	4.58
Pyr	4	$C_{16}H_{10}$	202	0.14	5.18	9.40	4.58
BaA	4	$C_{18}H_{12}$	228	0.01	5.91	10.6	5.30
Chr	4	$C_{18}H_{12}$	228	BDL	5.86	10.7	5.30
BbF	5	$C_{20}H_{12}$	252	BDL	5.80	10.8	5.75
BkF	5	$C_{20}H_{12}$	252	BDL	6.00	11.5	5.74
BaP	5	$C_{20}H_{12}$	252	BDL	6.04	13.6	6.74
IcdP	5	$C_{22}H_{14}$	276	BDL	7.00	11.7	6.20
DahA	6	$C_{22}H_{12}$	276	BDL	6.75	13.3	6.52
BghiP	6	$C_{22}H_{12}$	278	BDL	6.5	13.1	6.20
Me-PAHs							
2-MNAP	2	$C_{11}H_{10}$	142	40.6	3.86	5.53	3.47
1-MNAP	2	$C_{11}H_{10}$	142	40.6	3.87	5.54	3.48
2,6-DMNAP	2	$C_{12}H_{12}$	156	14.5	4.26	5.89	3.68
2,7-DMNAP	2	$C_{12}H_{12}$	156	14.8	4.26	5.89	3.68
1,3-DMNAP	2	$C_{12}H_{12}$	156	11.9	4.26	6.00	3.69
1,6-DMNAP	2	$C_{12}H_{12}$	156	11.5	4.26	6.02	3.69
1,4-DMNAP	2	$C_{12}H_{12}$	156	13.2	4.26	5.95	3.70
1,5-DMNAP	2	$C_{12}H_{12}$	156	12.9	4.26	6.22	3.61
1,2-DMNAP	2	$C_{12}H_{12}$	156	14.5	4.26	5.89	3.70
2-MPHE	3	$C_{15}H_{12}$	192	0.26	5.15	7.49	4.52
2-MANT	3	$C_{15}H_{12}$	192	0.19	5.00	7.63	4.51
1-MANT	3	$C_{15}H_{12}$	192	0.24	4.89	7.52	4.52
1-MPHE	3	$C_{15}H_{12}$	192	0.17	5.08	7.77	4.53
9-MANT	3	$C_{15}H_{12}$	192	0.17	5.07	7.70	4.53
3,6-DMPHE	3	$C_{16}H_{14}$	206	0.07	5.44	8.03	4.73
2,3-DMA	3	$C_{16}H_{14}$	206	0.07	5.44	8.03	4.73
9,10-DMA	3	$C_{16}H_{14}$	206	0.04	5.69	8.28	4.76
9-MMHEN	3	$C_{20}H_{16}$	256	0.01	5.82	8.95	5.84
2-MFLU	4	$C_{17}H_{12}$	216	0.05	5.48	8.90	5.06
1-MPYR	4	$C_{17}H_{12}$	216	0.05	5.48	6.90	5.06
1,2-MBaA	4	$C_{19}H_{14}$	242	0.01	6.07	9.71	5.57
7,9-MBaA	4	$C_{19}H_{14}$	242	0.01	6.07	9.71	5.57
4,6-MBaA	4	$C_{19}H_{14}$	242	0.01	6.07	9.71	5.57
3,5-MBaA	4	$C_{19}H_{14}$	242	0.01	6.07	9.71	5.57
10-MBaA	4	$C_{19}H_{14}$	242	0.01	6.07	9.71	5.57
5,8-DMBcPH	4	$C_{20}H_{16}$	256	BDL	6.62	10.2	5.81
6,8-DMBaA	4	$C_{20}H_{16}$	256	0.01	6.62	10.2	5.80
3,9-DMBaA	4	$C_{20}H_{16}$	256	0.01	6.62	10.2	5.78

7,12-DMBaA	4	$C_{20}H_{16}$	256	0.01	6.62	9.40	5.81
3-MCHA	3	$C_{21}H_{16}$	268	BDL	6.42	10.3	6.11
3,9-MCHA	3	$C_{21}H_{16}$	268	BDL	6.62	10.2	5.78
7,10-MBaP	5	$C_{21}H_{14}$	266	0.04	6.54	8.33	5.62
7,10-DMBaP	5	$C_{21}H_{14}$	266	BDL	7.2	11.5	6.33
NPAHs							
1-N	2	$C_{10}H_8O$	144	1130	2.69	8.48	3.48
2-N	2	$C_{10}H_8O$	144	151	2.69	8.65	3.29
1-NN	2	$C_{10}H_7NO_2$	173	1.25	0.79	14.4	3.95
2-NN	2	$C_{10}H_7NO_2$	173	1.25	0.79	14.4	3.95
2-NBP	2	C12H9NO2	199	15.9	3.57	7.74	3.91
4-NBP	2	$C_{12}H_9NO_2$	199	9.83	3.57	7.99	3.90
5-NAC	3	$C_{12}H_9NO_2$	199	888	1.98	13.9	4.14
2,2′-DBP	2	$C_{12}H_8N_2O_4$	244	962	1.19	17.3	3.79
9-NAN	3	$C_{14}H_9NO_2$	223	53.5	1.96	16.6	5.02
9-NPH	3	$C_{14}H_9NO_2$	223	53.5	1.96	16.6	5.02
2-NDB	3	$C_{12}H_7NO_2S$	229	0.30	4.11	9.86	4.11
3-NPH	3	$C_{14}H_9NO_2$	223	53.5	1.96	16.6	5.02
2-NAN	3	$C_{14}H_9NO_2$	224	53.5	1.96	16.6	5.00
9,10-DNAN	3	$C_{14}H_8N_2O_4$	268	17.6	1.78	18.8	5.10

PAHs, polycyclic aromatic hydrocarbons. Me-PAHs, Methylated polycyclic aromatic hydrocarbons. NPAHs, Nitrated polycyclic aromatic hydrocarbons. RN, Aromatic rings number. MW, molecular weight. Log Kow, logarithm octanol/water partition coefficient. Log KoA, logarithm octanol/air partition coefficient. Log Koc, logarithm organic carbon partition coefficient.

Table S5. Factor pattern of PCA for PAHs in 10 WWTPs, China.

**PAHs**	**PC1**	**PC2**	**PC3**
NaP	0.20	0.37	0.86
Acy	−0.07	0.78	0.46
Ace	0.39	−0.44	0.70
Flu	0.36	−0.80	0.42
Phe	0.81	−0.42	0.18
Ant	0.70	−0.64	0.00
Fluo	0.59	−0.68	0.16
Pyr	0.60	−0.75	−0.09
BaA	0.93	0.26	0
Chr	0.82	0.36	−0.32
BbF	0.93	0.23	−0.16
BkF	0.88	0.04	−0.41
BaP	0.92	0.22	−0.23
IcdP	0.37	0.69	0.26
DahA	0.66	0.65	0.08
BghiP	0.58	0.67	0.11
%Variance	44.9	30.6	13.4

PCA, Principal Component Analysis. PAHs, polycyclic aromatic hydrocarbons. WWTPs, wastewater treatment plants sites. PC1, PC1, PC1, represent Principal Component.

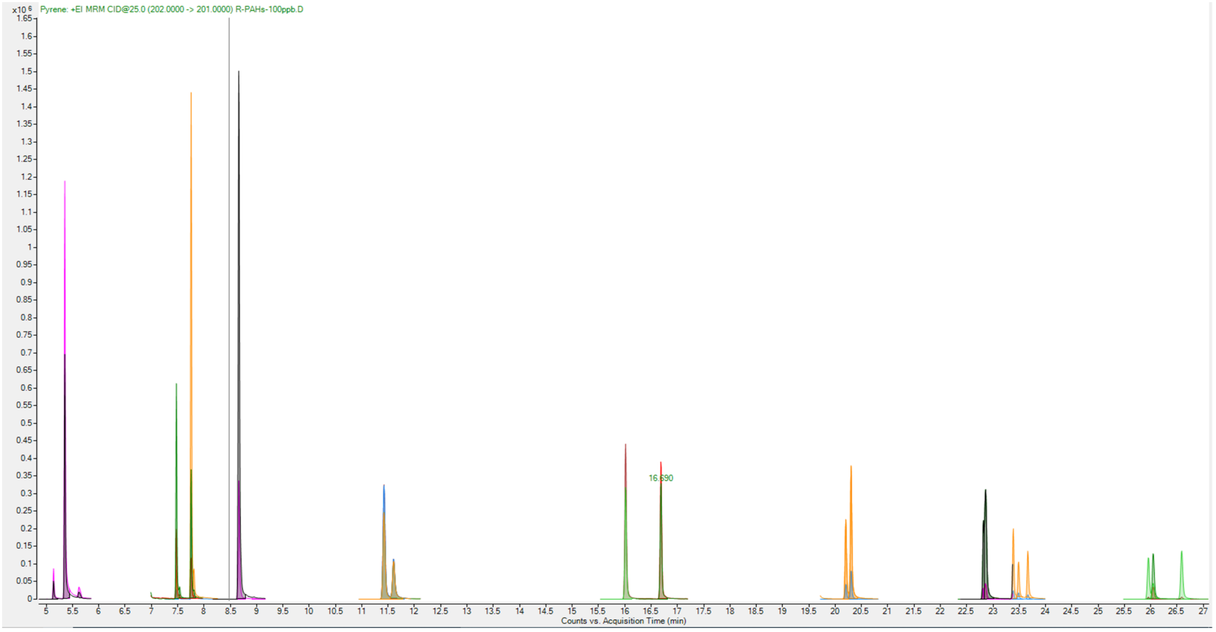

Figure S1. MRM chromatogram of target PAHs.

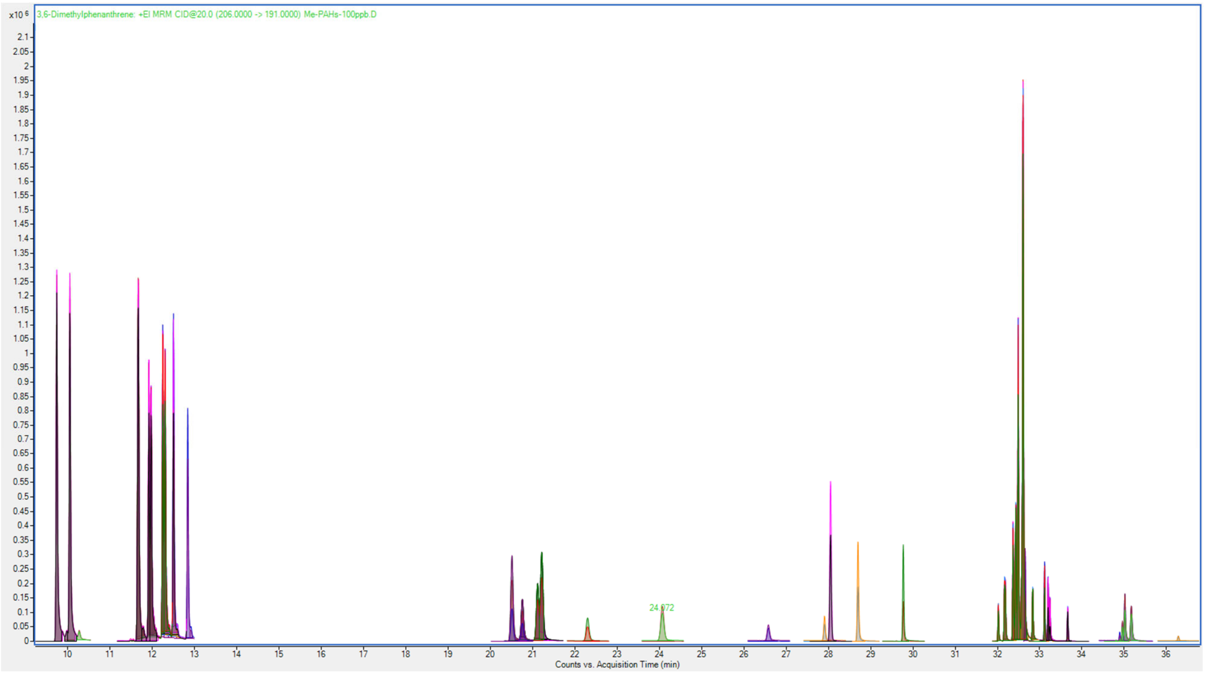

Figure S2. MRM chromatogram of target Me-PAHs.

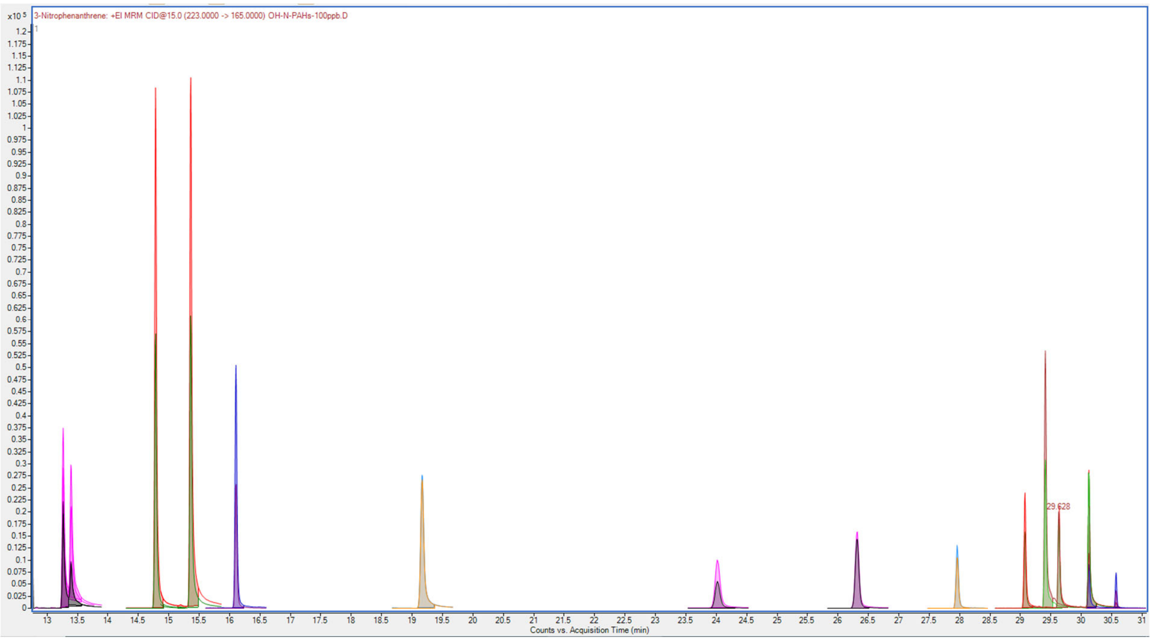

Figure S3. MRM chromatogram of target NPAHs.

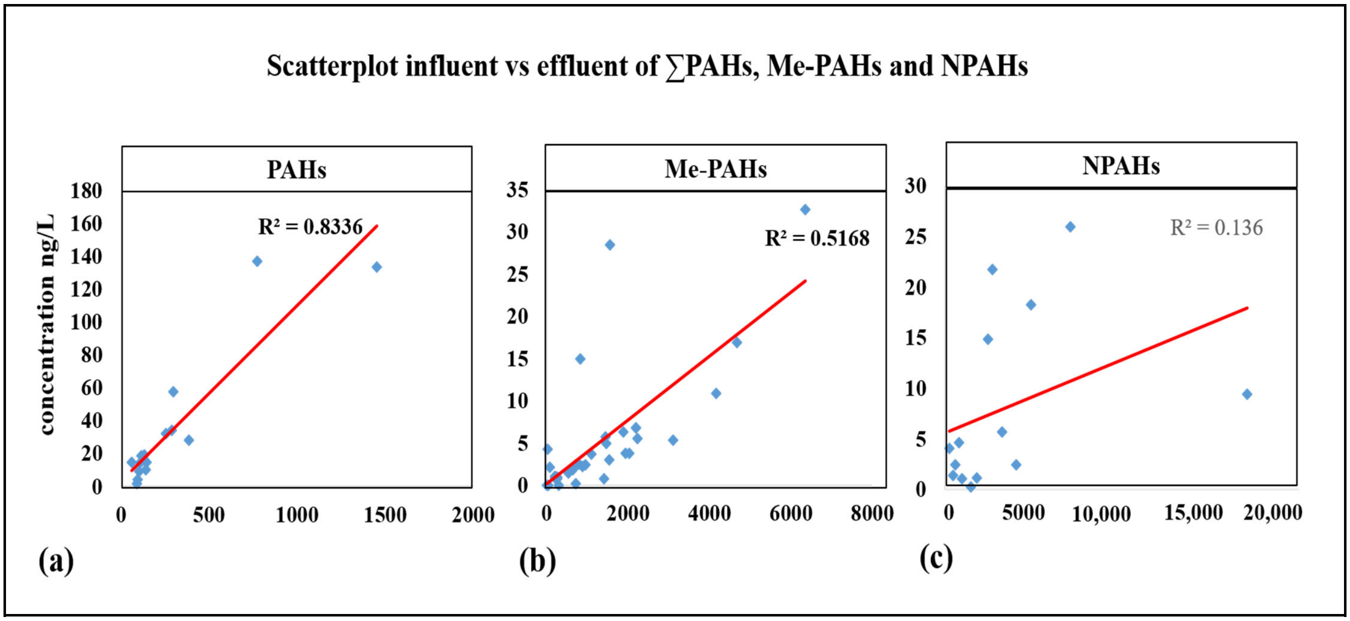

Figure S4. Scatterplot influent vs effluent of (**a**) ∑PAHs, (**b**) ∑Me-PAHs and (**c**) ∑NPAH.

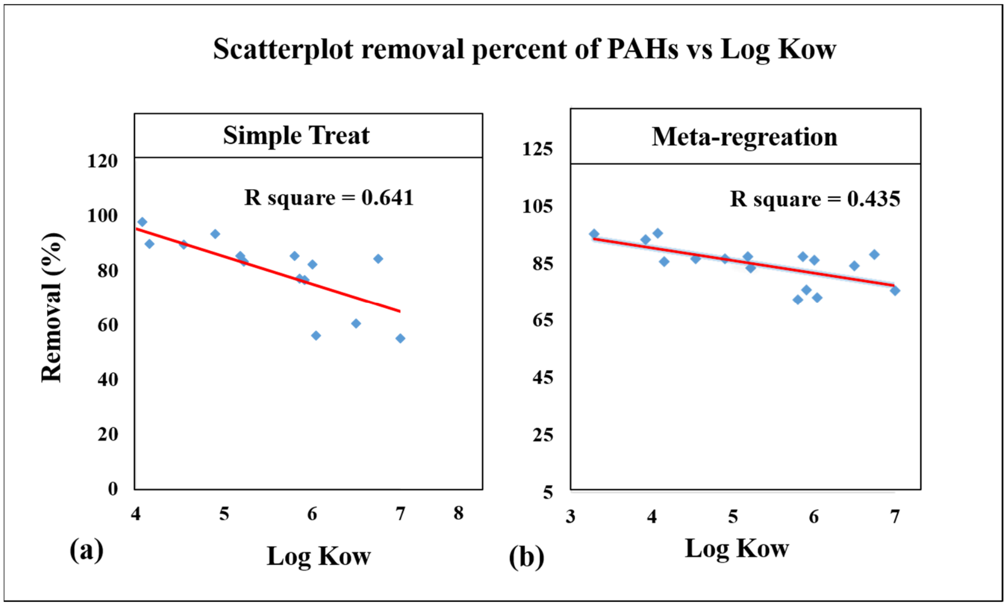

Figure S5. Percent removal efficiency of PAHs, Model Simple Treat (**a**) Model Meata-regression (**b**) versus Log Kow.

References

1. Paris, A.; Ledauphin, J.; Poinot, P.; Gaillard, J.-L. Polycyclic aromatic hydrocarbons in fruits and vegetables: Origin, analysis, and occurrence. *Environ. Pollut*. **2018**. *234*, 96–106.
2. Bandowe, B.A.M.; Meusel, H. Nitrated polycyclic aromatic hydrocarbons (nitro-PAHs) in the environment–A review. *Sci. Total Environ.*, **2017**. *581*, 237–257.
